# Direct and indirect healthcare and carbon savings with ACTIVE Particle Control^TM^ air-purification

**DOI:** 10.3389/fpubh.2022.1073858

**Published:** 2023-01-04

**Authors:** Mark Ereth, Judith Fine, Bency Massinello, Heather Gallagher, Eddie Simpser, Frank Stamatatos

**Affiliations:** ^1^Department of Anesthesiology, Mayo Clinic College of Medicine, Rochester, MN, United States; ^2^SecureAire Technologies, LLC, Dunedin, FL, United States; ^3^St. Mary's Healthcare System for Children, Bayside, NY, United States

**Keywords:** healthcare associated infections, healthcare expenditures, air purification, indoor air quality, carbon reduction

## Abstract

Controlling airborne transmitted disease remains a challenge to clinicians, healthcare administrators, and engineers. Engineering measures are critical to any infection control program but can require extensive installation procedures, may be expensive to maintain, and may not always demonstrate clinical or financial benefit. We determined the financial and carbon benefits of an engineering solution to combat air pollutants and to control airborne transmitted disease. We determined the costs of healthcare associated infections (HAIs), and the costs of installation, maintenance, energy demands, and carbon impacts of an ACTIVE Particle Control^TM^ (APC) air-purification system. In a 20 month study with over 65,000 patient days the significant reductions in HAIs resulted in significant financial, energy, maintenance, and carbon savings from this engineering solution. Positive clinical and financial outcomes are possible with novel air-purification solutions such as APC.

## 1. Introduction

**Figure 1:**
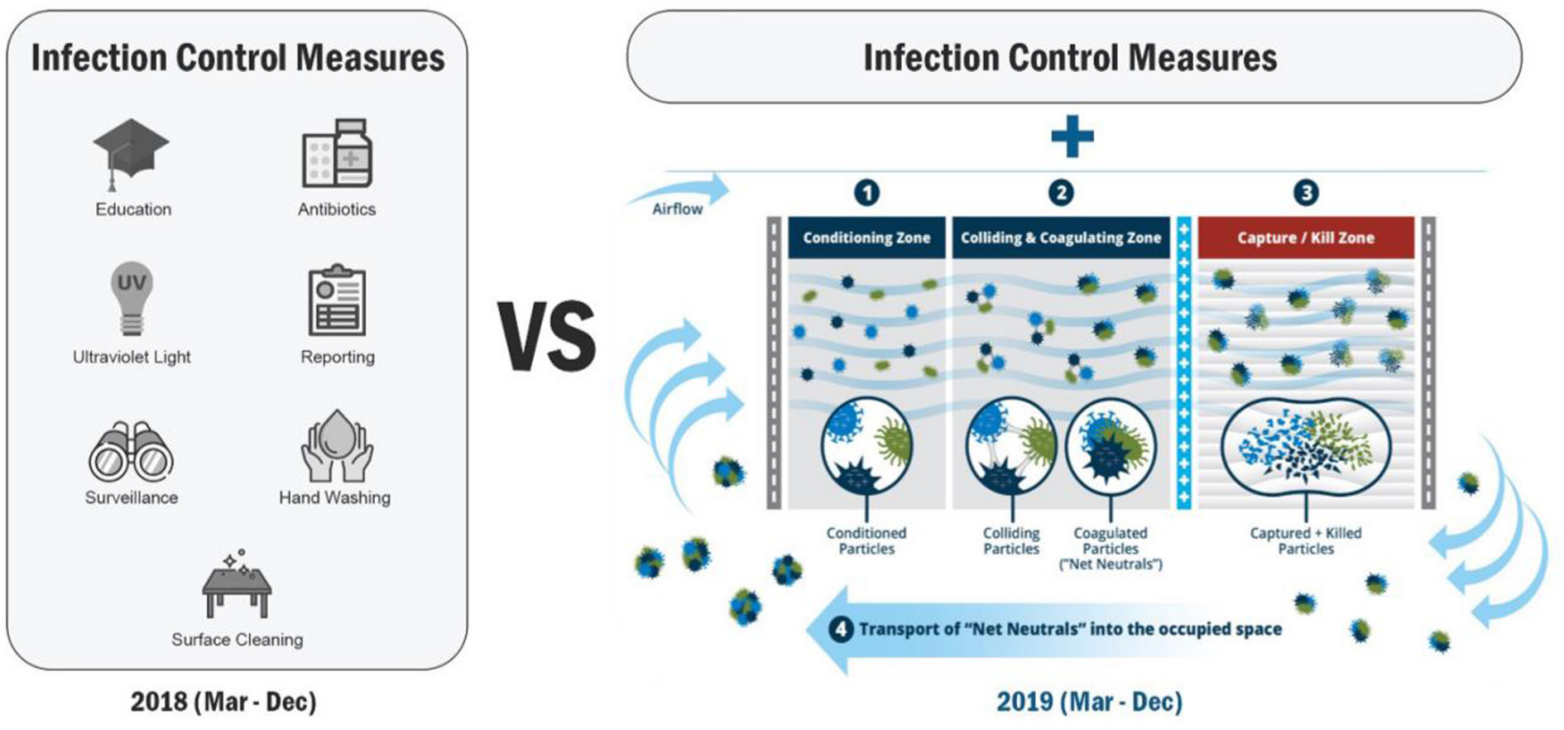
Study Methods: Comprehensive infection control measures included education and training, hand washing, isolation, surveillance and reporting, antibiotics, and selective ultraviolet light use in the index year (2018). All measures continued during the intervention year (2019) with the addition of ACTIVE Particle ControlTM (APC) technology. APC Mechanism of Action: ACTIVE Particle Control^TM^ (APC) technology works by local electromagnetic field manipulation [controlled ionization, enhanced polarization, and controlled particle transport (direction and velocity)]. These forces condition microscopic (10–2,500nm) particles and pathogens (1), so they continuously initiate millions of particle-particle (ionization) and particle-molecular (polarization) collisions (2). These collisions lead to immediate and permanent ionically driven aggregations of fine and ultrafine particles and pathogens into larger particles. With the larger aggregates attaining a critical mass, their transport becomes controlled by airflow and they can be carried by air currents to the particle collector where any biologic material is killed or inactivated (3). Lastly, net-neutral particles recirculate within in the ventilated space repeating the cycle.

Documenting the safety and efficacy of procedures and techniques in healthcare has always been important. Healthcare associated infections (HAI's) are infections that occur while receiving care in a hospital or other health care facility which occurs 48 h or more after admission or within 30 days of receiving healthcare (1). In the US 1.7 million patients acquire HAI's from bacterial, viral, or other pathogens. HAI's cost US health care over $30 Billion each year and kills nearly 100,000 patients each year (2). Multi-modal methods to prevent HAI'S include education, surveillance, and contact and hand-cleansing efforts. The SARS-CoV-2 pandemic has forced us to examine the ability of real-world ventilation and air purification strategies to prevent airborne transmission of disease-causing pathogens. Often cost-benefit technology evaluations are computer modeled or are conducted within highly controlled experimental chambers. The ideal cost-benefit determination of a new technology will include clinical outcomes and real-world measurements. Healthcare economics requires measuring the cost-effectiveness of clinical interventions including the financial impact of building, environmental, and engineering technology. The ideal engineering solution provides both clinical and financial benefits to a healthcare facility and the patients it serves. ACTIVE Particle Control^TM^ (APC) (SecureAire, LLC, Dunedin, FL) has been shown to reduce bacterial contamination and HAI's, reduce fine and ultrafine airborne particles and pathogens in operating rooms, reduce bacterial contamination in hospital compounding pharmacies, and rapidly inactivate or kill the highly resistant anthrax surrogate *Bacillus subtilis* (3, 4). This novel technology works by local electromagnetic field manipulation [controlled ionization, enhanced polarization, and controlled particle movement (direction and velocity)]. These forces condition the microscopic (10–2,500 nm) particles and pathogens within a space, so they continuously initiate millions of particle-particle (ionization) and particle-molecular (polarization) collisions. These collisions lead to immediate and permanent ionically driven aggregations of fine and ultrafine particles and pathogens into larger particles. With the larger aggregates attaining a critical mass, their movement becomes controlled by airflow and they can be carried by air currents to the particle collector (Figure 1).

Focusing on the impact of airborne transmission of HAI's is more important than ever. And the principles of source control, ventilation, filtration, and air disinfection are critical in the management of airborne infections especially in healthcare facilities (5).

Increased energy demands are typically associated with higher performing filter media such as HEPA filters. Increased filter resistance across the media continuously increases the fan motor energy demands to move air against the increased pressure drop across the filter. Reduced energy demands can occur when the filter performance is enhanced in the absence of a tighter filter mesh. APC and similarly designed technology with electronic enhancement of filter performance have reduced energy demands (6).

We know of no previous studies using APC, or other ventilation or fitlrations strategies where an enitre facility, multiple buildings, have been treated with an ubiquitous engineering solution and real-world clinical outcomes have been measured over months or years.

When examining the cost-effectiveness of an engineering solution within a healthcare facility the clinical, financial, energy, and carbon impacts should all be considered. We sought to determine the financial and energy-saving benefits of a ubiquitous air purification solution within a healthcare facility.

## 2. Methods

The infection control records, clinical census, and HAI associated expenses were reviewed from the St. Mary's Healthcare System for Children for the years 2017 through 2022. Specific HAIs identified included respiratory viral pathogens, conjunctivitis, tracheitis, cellulitis, urinary tract infections, pneumonia, and sepsis. The complete data on the direct costs associated with conjunctivitis, tracheitis, cellulitis, urinary tract infections, pneumonia, and sepsis were difficult to obtain or incomplete and thus eliminated from this analysis. For the purpose of this study, we only examined the impact of APC on the most common HAI in this facility; respiratory viral pathogens that included Enterovirus, Rhinovirus, Adenovirus, Parainfluenza virus, Human metapneumovirus, and Coronavirus (Pre/Not SARS-CoV-2).

The APC system was installed and three roof-top air-purification APC units served all but one patient ward of this 124-bed pediatric post-acute care facility. They were fully operational by February 1, 2019. Pre-installation data was examined for March-December 2018. Post-installation data was examined for March-December 2019 (Figure 1). Data from January and February 2019 was considered mixed between control and intervention periods and was thus eliminated. Data from the un-treated patient ward was excluded from analysis. The direct, indirect, average variable, and acute care hospitalization costs for the HAIs were determined from internal and external sources (7–9). Direct energy demands and carbon impact were measured and calculated before and after the installation of the APC system based on filter pressure-drop specifications, fan-motor energy requirements, local utility rates, and motor-blower efficiency (Supplementary Figure 1) (10, 11). Carbon equivalency was determined with an Environmental Protection Agency (EPA) carbon converter (11).

All data were collected, analyzed, and reported by the clinical, engineering, and finance-analytics personnel at St. Mary's Healthcare System for Children. The mean, median and interquartile ranges were determined for all HAIs including respiratory viral illness. Comparisons were made between the clinical, financial, energy, and carbon outcomes for the standard infection control program before and after the addition of the APC system. Comparisons between these three groups were conducted using *t*-tests, analysis of variance, pairwise Bonferroni multiple comparisons and *R*^2^ analyses. *P* < 0.05 was considered to indicate statistical significance (< 0.017 for Bonferroni correction).

## 3. Results

The addition of APC to standard infection control measures was associated with a 43.7% reduction in HAI's (from 11.9/1,000 patient days to 6.7/1,000 patient days) *p* < 0.0001. This represented significant reductions in respiratory viral pathogens, tracheitis, conjunctivitis, and cellulitis (3). There were non-significant but small to moderate reductions in pneumonia, sepsis, and urinary tract infections. Of those patients diagnosed with respiratory viral pathogens, ~8% required an acute care admission. For those patients briefly transferred for higher acuity care, the internal direct costs of care and the real-world loss of revenue to the hospital from a clinical unit lockdown were included. The annual direct savings to the St. Mary's system for reduced respiratory viral pathogens was $363 for each of 22 cases of reduced complications ($7,986). The discounted and retained revenue for St. Mary's Healthcare from reduced clinical unit lockdowns was 5 days each year or $10,045 annually.

**Figure 2:**
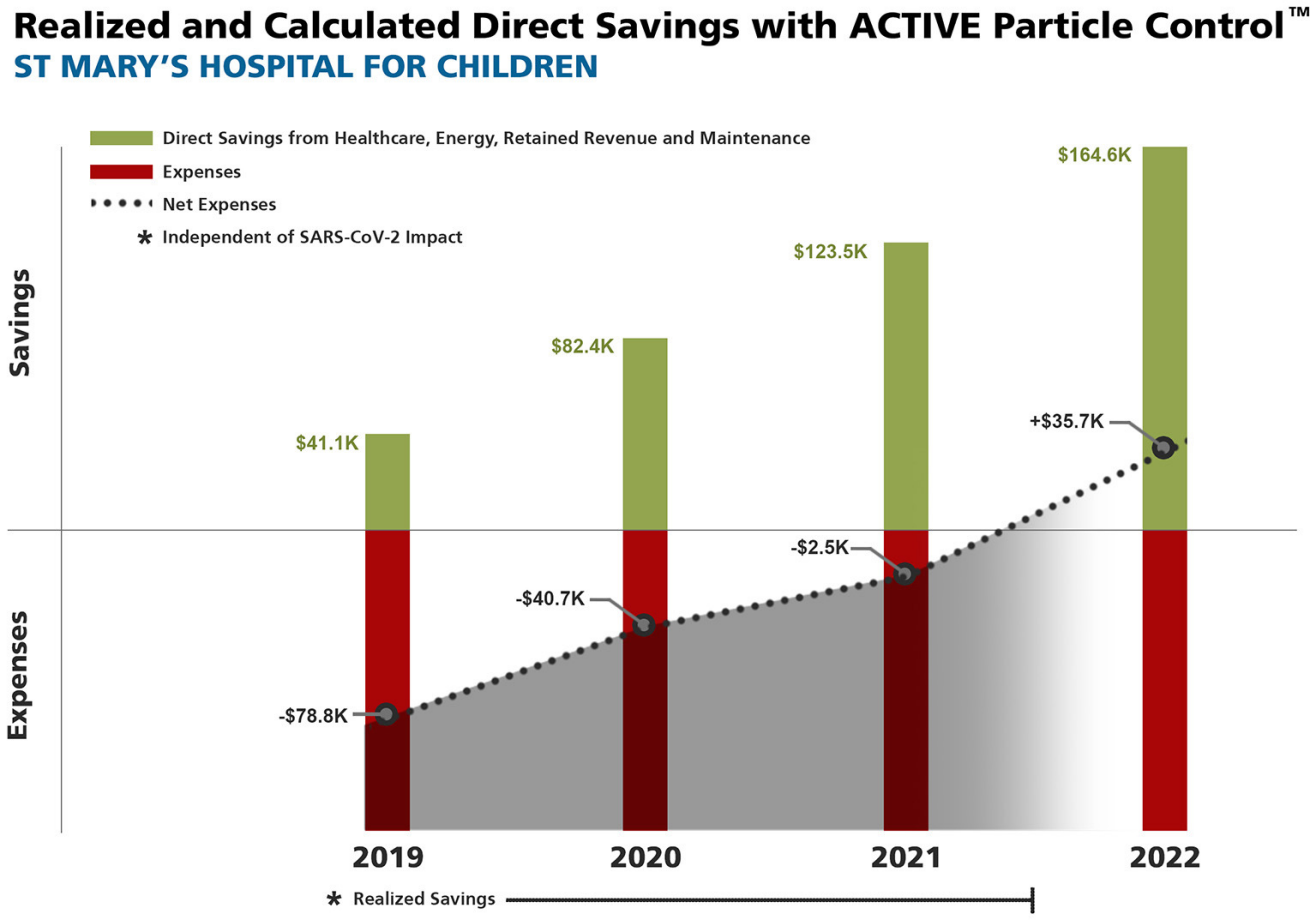
Realized and calculated Cumulative Direct and Net Savings from healthcare-associated infection (HAI) avoidance, energy and maintenance savings, and retained revenue with ACTIVE Particle Control^TM^ (APC) technology. All HAI data collected prior to COVID-19. *Does not include broader additional healthcare system savings of $25,592 each year, nor the annual 52 ton reduction in carbon dioxide generation.

**Figure 3:**
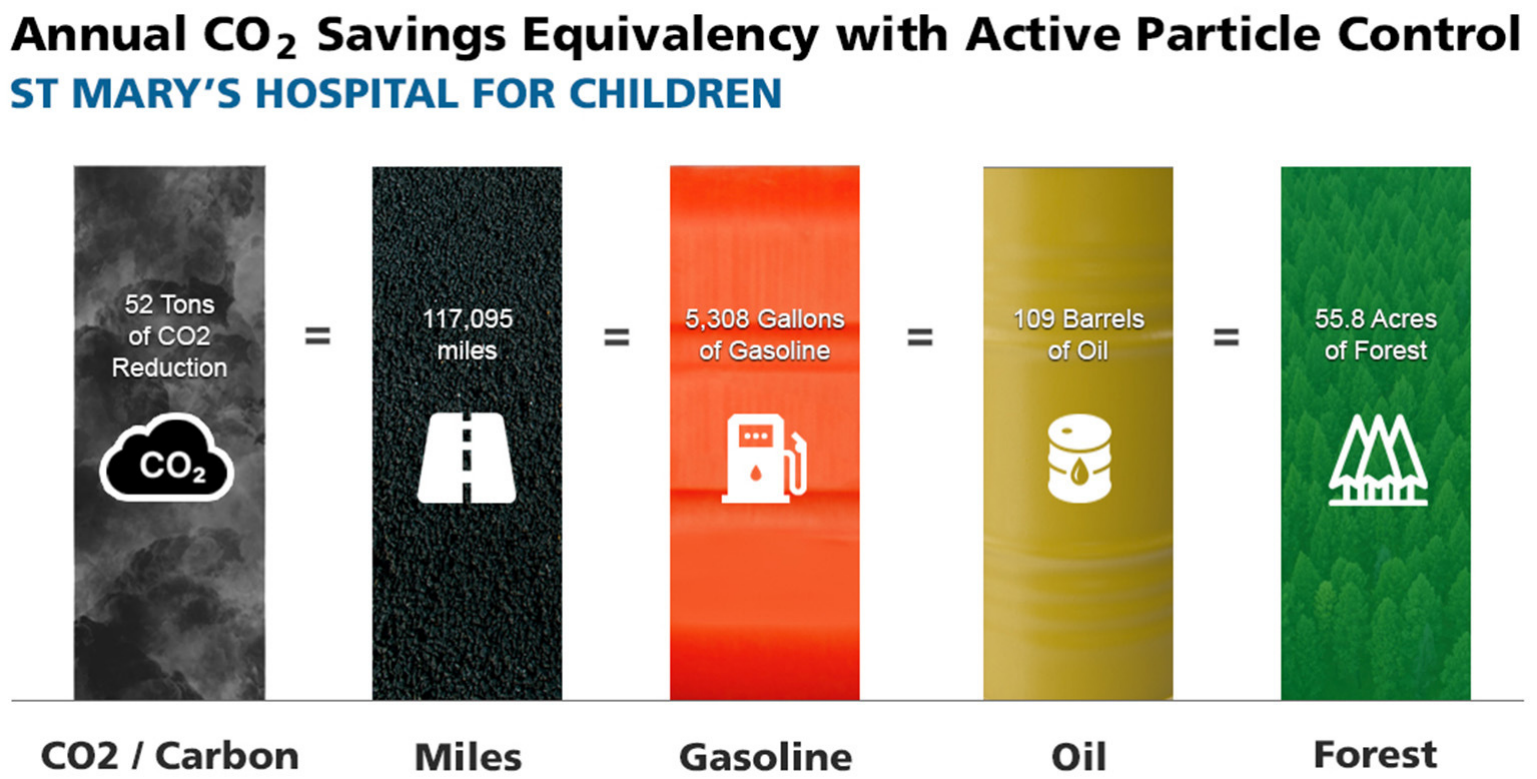
Annual carbon savings from the deployment of ACTIVE Particle Control^TM^ (APC) technology. The 51.9 ton reduction in annual carbon dioxide (CO_2_) generation is equivalent to 117,095 miles driven with a typical passenger car, the consumption of 5,308 gallons of gasoline, the consumption of 109 barrels of crude oil, or the carbon sequestered by 55.8 acres of United States forests (8). All data collected prior to COVID-19.

The initial and annual cost of installation and maintenance of the APC system for the first 2 years totaled $123,000. The annual maintenance costs of the APC filter media were less than the previously used MERV 15 filters and provide an annual savings of $2,976. The gross and net financial data on cumulative expenses and savings are presented in Figure 2 and Supplementary Figure 2. The annual energy savings were 79,219 kilowatt hours (kWh) and based on local New York City utility rates resulted in $16,162 in utility savings. The significant annual reduction in carbon dioxide generation was 103,896 pounds (or >51.9 tons) (*p* < 0.01). The annual carbon savings was equivalent to the impact of a 55.8 acre US forest (Figure 3).

## 4. Discussion

The installation of a facility-wide airborne infection control and air-purification engineering solution added to standard infection control measures was associated with significantly reduced HAI costs, energy and maintenance costs, and CO_2_ generation.

Examining the clinical impact of an engineering solution to combat infections is complex. Even more difficult is then determining the financial impact of those clinical results. All HAI data presented are from immediately before the SARS-CoV-2 pandemic and thus not impacted by any pandemic-related restrictions or interventions. This patient population had a large number of co-morbidities that placed them at higher-than-normal risk of infection. Thus, the results of the intervention may or may not be greater than they would be in a general hospital (or traditional healthcare facility). The current work was tightly controlled but like all clinical studies we could not account for every possible variable.

Aside from the introduction of the APC technology the facility infection surveillance and control measures remained stable throughout the study period. This real-world study was conducted by internal personnel who collected internal data from St. Mary's Healthcare System for Children over a 20-month period that included over 65,000 patient days. Further, we did not include the financial benefits of avoiding tracheitis, conjunctivitis, and cellulitis as precise data on these complications were difficult to obtain. Any such financial benefit here would add to the overall financial benefit of the APC technology.

Typically, the evaluation of engineering solutions for air-purification and airborne infection control are conducted in highly controlled small room or experimental chambers.

Engineering solutions such as ultraviolet light and dry hydrogen peroxide have resulted in reduced pathogen colonization of surfaces but there is limited information on the impact on clinical infections in real-world settings (12, 13).

Recently an exhaustive review of studies on anti-infection procedures revealed they were based more on laboratory data rather than *in situ* efficacy (14). The authors advised practitioners and researchers to consider system-level efficacy and not just the efficacy of a focal setting. Even with numerous technical and engineering solutions being deployed during the pandemic there are few real-world studies conducted over years on 50,000 or more patient days on other engineering solutions. Higher outdoor ventilation likely reduces airborne pathogen exposures and HEPA filtration has demonstrated clinical impact on asthmatic symptoms. Yet there are few long-term and real-world evaluations of population based *clinical outcomes* with various technologies. Ultraviolet light, photocatalytic electrochemical oxidation, and bipolar ionizers have demonstrated efficacy in laboratory, experimental chambers, or small rooms. Yet to our knowledge there also remains a lack of findings demonstrating a reduction in clinical infections in real world settings. In addition, bipolar ionizers have been associated with the generation of toxic compounds and are under scrutiny by the EPA (15). Ratliff et al. (16), just published an evaluation of bipolar ionization and photocatalytic devices in a large-scale testing facility. They report little to no benefit of either technology in reducing particle counts or inactivating microorganisms and in-fact demonstrated significant ozone generation with photocatalytic technology. This underscores the need for more practical investigation of accepted and novel technical solutions such as APC (14).

Beyond the clinical and financial benefits of this engineering solution to combat infections were non-tangible but important benefits for patients and their families. Numerous patients were able to be weaned off of mechanical ventilation and were able to be extubated and return to normal spontaneous ventilation due to the significant reduction in facility infectious load (Personal Communication, Edward Simpser, MD). The downstream impact on a child's life by not requiring mechanical ventilation meant better speaking and communication, which leads to better education, socialization, and independence. The impact on the lives of such patients is not easily measured in dollars or cost savings, yet they are significant.

As per New York State regulations and CDC outbreak protocols, no admissions to the units during “lockdown procedures” occur whenever an infectious “outbreak” occurred. An “outbreak” is when three or more residents infected with the same pathogen are placed on infections precautions for a proscribed time and new admissions are prohibited. As a direct result of the “lockdown” protocols hospital personnel did not admit to any open beds in the unit until the outbreak/lockdown had resolved. The installation of APC was associated with a reduction in outbreaks and a reduction of unit lockdowns. The attributable direct savings from reduced lockdowns was based on clinical and population determinants.

An additional indirect benefit that could not be accurately determined was the reduction in costs to the broader healthcare system. The annual non-St. Mary's cost savings for those patients who were transferred for acute care was ~$25,592 to the broader healthcare system. These indirect savings are only an estimate and thus were not included in the summary results.

While the energy savings are direct, the decarbonization impact is less tangible and indirect but non-etheless a clear benefit. According to the EPA carbon calculator the reduced CO_2_ emissions that resulted from the APC installation is equivalent to 55.8 acres of forests sequestering carbon for a year (8).

These data demonstrate a positive financial impact from the deployment of APC in addition to the previously reported positive clinical impacts (3). In addition to clinical benefits of APC, the installation of this system resulted in energy savings and reduced carbon impact due to reduced fan-motor energy demands (7). With the addition of energy savings, the direct and indirect financial and carbon impact with the installation of an ubiquitous facility-wide air-purification system provided a significant financial return. Infectious and energy savings will vary between facility, patient characteristics, clinical procedures, and local utility rates. The direct savings to St. Mary's Hospital for Children and the patients and families it serves from reduced infections, energy savings, reduced maintenance costs, and retained income provided significant financial benefits after the installation of APC.

A direct comparison of the clinical infection outcomes between various engineering solutions such as HEPA filtration, fresh air ventilation rates, ultraviolet light, photocatalytic electrochemical oxidation, bipolar ionizers, and dry hydrogen peroxide in real-world settings is needed.

## 5. Conclusion

In conclusion, a novel facility-wide airborne infection-control and air-purification system added to standard infection control procedures resulted in significant financial benefits from the reduction in HAIs, and reduced energy requirements that significantly reduced the facility carbon footprint.

## Data availability statement

The raw data supporting the conclusions of this article will be made available by the authors, without undue reservation.

## Author contributions

ME was responsible for manuscript preparation and coordination. JF, BM, and HG were responsible for data collection, review, and analysis. ES was responsible for study, administrative, financial oversight, and manuscript review. FS developed the energy cost calculation tool. All authors contributed to the article and approved the submitted version.
