# Supplementary material for: Direct and indirect healthcare and carbon savings with ACTIVE Particle ControlTM air-purification
Source: Front Public Health. 2023 Jan 4;10:1073858. doi: 10.3389/fpubh.2022.1073858 (PMC9845911; doi:10.3389/fpubh.2022.1073858)

Supplementary Material

Supplementary Figure 1. **EPA energy-carbon equivalency calculator.**
https://www.epa.gov/energy/greenhouse-gas-equivalencies-calculator#results

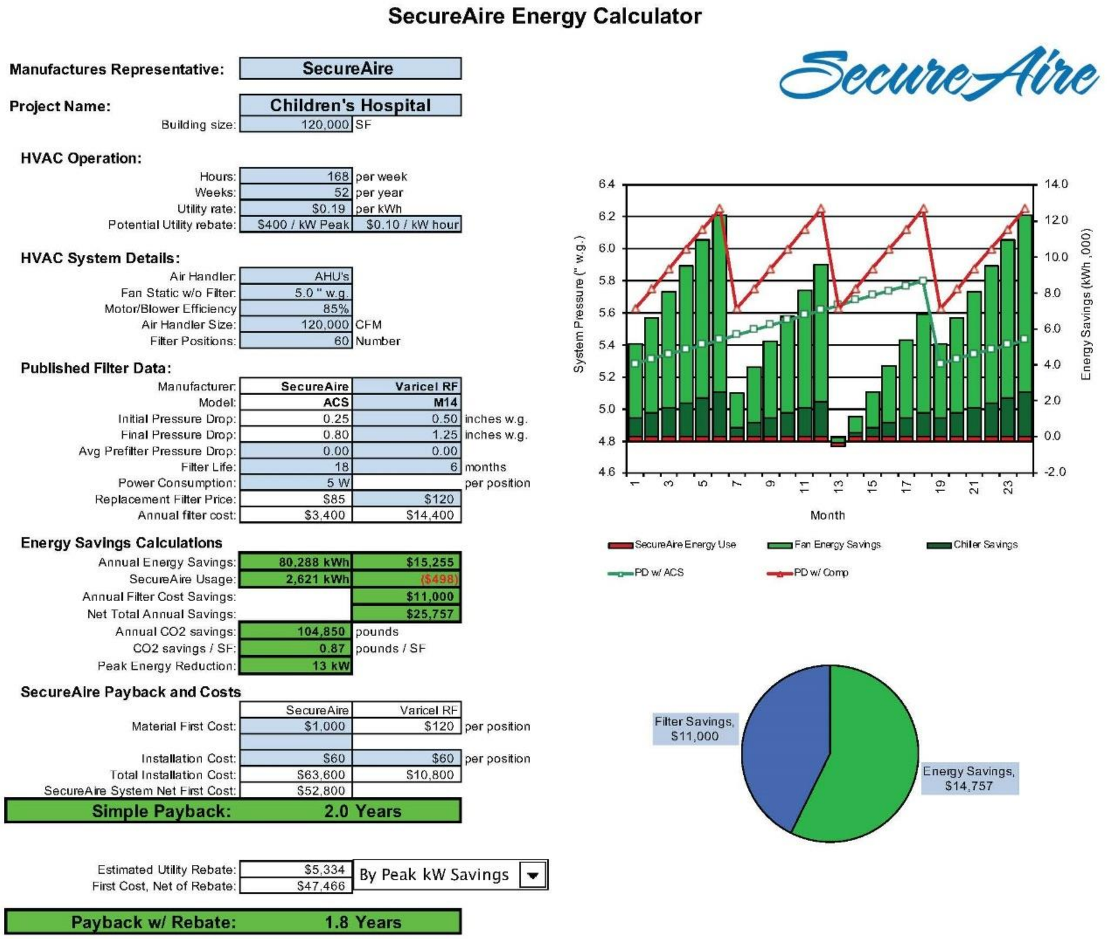

Supplementary Figure 2. Cumulative Direct Savings from healthcare-associated infection (HAI) avoidance, energy and maintenance savings, and retained revenue with ACTIVE Particle Control^{TM} (APC) technology. Realized savings for 2019, 2020, and 2021. Calculated savings for 2022. All data is independent of COVID-19 impact.

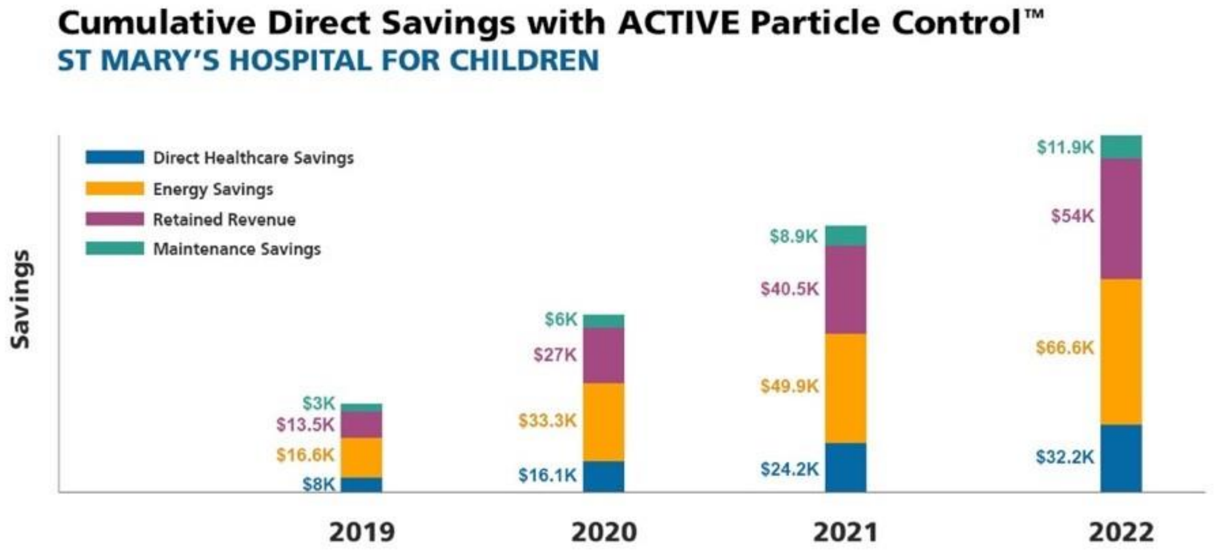

Supplement: Supplementary file 1 [file Data_Sheet_1.PDF]
